# Informal care: choice or constraint?

**DOI:** 10.1111/scs.12441

**Published:** 2017-04-12

**Authors:** Hareth Al‐Janabi, Fiona Carmichael, Jan Oyebode

**Affiliations:** ^1^ Health Economics Unit Institute of Applied Health Research University of Birmingham Birmingham UK; ^2^ Business School University of Birmingham Birmingham UK; ^3^ Faculty of Health Studies University of Bradford Bradford UK

**Keywords:** informal care, choice, motivation, well‐being, quality of life, survey

## Abstract

**Background:**

‘Choice’ is increasingly pursued as a goal of social policy. However, the degree to which choice is exercised when entering an informal caring role is open to debate.

**Aim:**

In this study, we examined the degree of choice and constraint in entering a caring role, and the relationship between choice and carers’ well‐being.

**Methods:**

Data were derived from 1100 responses to a postal survey conducted in a British city. Statistical tests of association and multivariable regression modelling were applied to study the factors associated with choice in entering a caring role and the association that choice in entering a caring role had with carers’ well‐being.

**Results:**

We found that informal care was generally perceived to be a free choice, albeit in most cases, a choice was also constrained by duty, financial or social resources. Having a sense of free choice in entering care was strongly and positively associated with the carer's well‐being.

**Conclusion:**

The study findings are consistent with a view that enabling individuals to have more choice in their caring roles may be beneficial.

## Introduction

Rising healthcare costs means that governments increasingly look to the family for care for individuals unable to look after themselves 1, 2. In the UK, 2011 Census figures suggested that over six and a half million people, just over 10 per cent of the population, were involved in family (informal) care of an adult or disabled child 3. In total, 38 per cent of carers in England and Wales were estimated to provide 20 or more hours of care a week with 23 per cent providing 50 or more hours of informal care 3. Informal care can be very demanding, often requiring individuals to sacrifice their own health 4, work 5 and relationships 6. While many people willingly care for a loved one at times of need, the degree to which they exercise a choice in doing so is open to debate. Given the increased attention to choice for care recipients in policymaking 7, it is worth examining the degree to which providing informal care is perceived to be free choice by the individuals concerned.

The focus in this study is on choice in taking on the caring role. The issue of degree of choice within the caring role, for example in relation to combining work and care, is discussed elsewhere 8. Choice on entry to a caring role refers to the degree to which the carer has a sense of freedom about whether to opt into the role. A perceived lack of choice could be related to particular social or environmental constraints. In contrast, the conscious exercising of choice to take on a caring role could be related to one or more motivations for caring.

The reasons why so many individuals decide to engage in informal care, particularly when it is very time intensive, are not well understood. At one end of the spectrum, there is the view that people who become carers do so because they feel obliged to act as a carer when a family member becomes ill 9, 10, 11. This sense of responsibility or duty is tied to social norms 12 and may allow little room for manoeuvre. For example, a social norm that children should care for their ageing parents, as legally formalised through the French *obligation alimentaire* system, would oblige people to provide elder care, although this could be shared amongst siblings or one or more siblings could take overall responsibility. The responsibility view suggests that demand will create its own supply and this perspective is supported to some extent by evidence that the proportion of women providing intensive (>15 hours per week) out‐of‐home care in the USA is identical across prebaby boomer and baby boomer cohorts 13.

Carers may be constrained by factors other than duty. Carers are often in poor financial circumstances 14; this may limit private care options and inhibit carers’ ability to exercise choice about entering caring. There may also be few, if any, other care options within the family. This may arise as a result of a physical absence of other family members to care or because certain members of the family are unwilling to take on a caring role 15, 16.

At the other end of this spectrum, the view is that a decision to undertake care is essentially a rational choice. This decision reflects individual cost benefit calculations linked to factors such as loss of income, ill health or increased stress due to caring responsibilities and any avoidance of guilt, satisfaction or ‘process utility’ derived from the provision of informal care 17. The rational choice perspective is supported by evidence that individuals in full‐time employment and higher earners are less likely to take on intensive caring responsibilities 18. The rational choice perspective suggests that whether or not the supply of informal care will increase to meet any rise in demand will depend on trends in employment and policies to support working carers.

A pragmatic approach would suggest that the degree of choice available to potential carers will differ depending on their individual circumstances and those of the people needing care; for some, the degree of choice may be more constrained than for others. The nature of these circumstances might also change over time and as people age. The critical feminist economics discourse on altruism, reciprocity and norms of responsibility provides some insights into these potential influences. Folbre (19, p.75) defines caring as ‘labour undertaken out of affection or a sense of responsibility for other people, with no expectation of immediate pecuniary reward’. The concept of reciprocity for either tangible or emotional services is linked to systems of gift giving 19 and precautionary expectations about an individual's own future care needs. Reciprocity implies the existence of a prior or extant relationship 20; for example, elder care by children reciprocates the original gift of the parent's care.

To date, there has been relatively limited empirical study of the degree to which family members feel they are exercising choice in taking on a caring role 8. One recent US study of older carers found under half of carers perceived their care to be a free choice 21. Similar findings have been reported in the UK, with the 2009/2010 Survey of Carers in the Household suggestion that many carers perceived little or no conscious choice in caring. In this study, 54% stated that caring was expected of them (i.e. it is what families do), 15% stated the care recipient would not want anyone else doing the caring and 12% reported that no one else was available 22.

The high proportion of carers who feel constrained in their caring role is underscored by related research on the reasons for caring. A study of Dutch carers found that the most common reason for caring was ‘duty’ and that other constraints such as being ‘the only one…available’ were also mentioned as important factors 17. Cicerelli found that caregiving was motivated by both a sense of obligation and a sense of attachment 23, and a stronger sense of obligation was associated with greater feelings of burden. In contrast, a Europe‐wide study of carers found ‘emotional bonds’ (i.e. love and affection) were the principle motivation for providing care 24. Duty, obligation and a lack of other alternatives were highlighted in far fewer cases. The importance of emotional bonds in motivating care and sustaining carer well‐being has also been found in the context of dementia care, where pre‐existing relationships characterised by reciprocity were associated with higher carer well‐being 25. Given the context for informal care, it can be seen that the constraints placed by normative and societal pressures, as well as necessity, are likely to limit objective choice to provide family care, but also that the anticipated rewards of caregiving may lead some to take on the role of carer out of choice.

The evidence on caring and well‐being suggests that, in general, more intensive caring roles, and specifically transitions into caring roles, are associated with lower levels of well‐being 26, 27, 28, 29. The negative effects on well‐being are especially strong for carers who are closely related to the care recipient 27. In terms of exercising choice about entering caring, Schulz et al. 21 found that a lack of choice amongst carers of older people was associated with greater emotional stress, physical strain and negative health effects. Exercising choice to enter a caring role may indicate that the carer thinks they will be able to handle the caring role. Indeed, it has been suggested that the negative impact of caring on well‐being may stem from the loss of autonomy and choice that an intensive caring role imposes 27. Furthermore, autonomy over one's life is seen as intrinsic to well‐being in self‐determination theory 30 and the capability approach 27. As such, it seems likely that greater choice to enter a caring role will be associated with higher levels of well‐being, whether the focus is on hedonic well‐being (indicated by traits such as happiness and life satisfaction) or eudemonic well‐being (indicated by traits such as capability and flourishing).

Our study addresses a gap by examining both choice and constraints in relation to entering caring. This is important because choice and constraints are likely to act simultaneously and may play a large role in the carer's subjective experience of caring. Our objectives were to (i) establish the degree to which individuals perceive caring to be choice or a constraint; (ii) identify whether the degree of choice varies according to carers’ characteristics and caring role; and (iii) estimate the association between choice in caring and the carer's well‐being. We investigated these issues using data from a survey of individuals living in a large city in the UK.

## Methods

This study is a secondary analysis of cross‐sectional data collected through a local government survey of residents’ quality of life. The survey covered a range of aspects of individuals’ lives, including a module of questions about the provision of informal care. The informal care questions covered the individual's decision to provide care; these survey data therefore offered an opportunity to study the decision to care amongst a heterogeneous group of carers in a community setting. Alongside data on informal care, data were collected on individuals’ socio‐demographic circumstances and well‐being, and these data were used to examine the factors associated with choice in caring and the relationship between well‐being and perceived choice in caring. Details of the survey, the construction of the choice in caring variable and the subsequent analysis are reported below.

### The quality‐of‐life survey

The data used in the study came from the 2009 Bristol City Council Quality of Life Survey 31. This is an annual postal survey of 25 000 representative residents of Bristol. Residents are randomly selected from the electoral register for this voluntary postal survey every September. The survey covers a range of topics relevant to the local authority, including the individual's local area, home, lifestyle, well‐being and socio‐demographic characteristics. In 2009, respondents were also surveyed about their informal care. To identify those providing informal care, respondents were asked, as part of the survey, whether they ‘looked after or gave any help or support to family members, friends, neighbours or others because of long‐term physical or mental ill health or disability, or problems related to old age’. The full question, provided in the Appendix S1, closely resembles the question used in the UK population census. Respondents who indicated that they provided informal care were asked to complete a set of additional questions about their informal care provision. These are abbreviated and listed below:

- How many hours of care are provided per week? (< 20 hours/20‐49 hours/50 hours +)
- How old is the care recipient? (0‐17 years/18‐64 years/65 years and over)
- What is the health of the care recipient like? (good/fair/bad)
- Are you the main carer for this person? (yes/no)
- Do you help with personal care? (yes/no)
- Do the following features of care provision apply?
  ○ I provide care because it is my duty? (yes/no)
  ○ I had a free choice to provide care? (yes/no)
  ○ There was no one else to provide care? (yes/no)
  ○ There was no money for paid care? (yes/no)

Respondents also completed the Carer Experience Scale (Al‐Janabi et al., 2008) ‐ a six‐item scale measuring carer quality of life. No questions were included on other aspects of informal care provision, for example, on the duration of caring, the carer–recipient relationship, the carer's marital status, whether they co‐resided with the care recipient or whether they had multiple caring roles.

5771 individuals responded to the survey, of whom 1 100 (19%) indicated that they provided some informal care in a typical week. This survey met the Local Authority standards of conduct, was compliant with UK law on data protection, and participation was both anonymous and voluntary.

### Creating a variable to indicate perceived ‘choice in caring’

We combined the responses to the four questions concerning individuals’ decision to care to simultaneously examine carers’ perceptions of choice and constraints in caring. This allowed for a more nuanced analysis than simply studying the responses to the four questions in isolation. To investigate the degree to which caring was perceived to be a free choice, carers were categorised into three groups. In the first group were carers who reported caring was a free choice, and not constrained by duty, finances or social support (‘free‐choice’ carers). A second group comprised those carers who reported caring was a free choice, but also reported that at least one of the constraints was also present (‘constrained choice’ carers). The third group comprised carers who indicated that caring was not a free choice (‘unfree’ carers).

### Predictors of choice in caring

The analysis involved two stages. The first investigation focused on identifying whether perceived choice in caring (as measured by the new three category variable) was affected by socio‐demographic factors and the nature of the caring role entered into. To examine the effect of socio‐demographic factors, we examined variables related to demography (age, sex, health status), culture (religion, ethnicity) and empowerment (qualifications, home ownership and receipt of benefits). For caring role, we examined variables related to the nature of caring role (primary or secondary), the provision of personal care, hours of caring, the health status of the care recipient and the age of the care recipient. We used cross‐tabulations to explore the magnitude of associations between perceived choice in caring and these variables and, given the ordinal nature of the perceived choice variable, used Kruskal–Wallis tests to identify statistical significance of any associations.

### Associations between choice in caring and carer well‐being

The second stage of the investigation focused on the relationship between perceived choice in caring and carers’ subsequent well‐being. We examined individuals’ hedonic well‐being 32 using two survey questions about their happiness and satisfaction with life. The happiness question had four possible responses on a Likert scale ranging from ‘not at all happy’ to ‘very happy’. The life satisfaction question was rated from 1 (‘completely dissatisfied’) to 10 (‘completely satisfied’). We examined individuals’ eudemonic well‐being 33 using the ICECAP‐O 34 capability questions and, for carers, additionally, the Carer Experience Scale 35. The ICECAP‐O measure comprises questions about five core capabilities in life, and responses can be scored to generate an overall score between 0 (no capability) and 1 (full capability) for the respondent 34. The Carer Experience Scale comprises questions about six aspects of care‐related quality of life, and responses can be scored to generate a score between 0 (worst caring experience) and 100 (best caring experience) 36. The life satisfaction and happiness questions are listed in the Appendix S1 along with the ICECAP‐O and CES items.

To analyse the relationship between the measures of well‐being and perceived choice in caring and well‐being, we used Kruskal–Wallis (nonparametric) tests. We then used multivariable regression modelling, to allow for the fact that the relationship between well‐being and perceived choice in caring may be confounded by other factors. This involved modelling well‐being responses as a function of the degree of choice in caring (‘free’, ‘constrained’ or ‘unfree’) controlling for socio‐demographic and care‐related factors. We estimated the regression model where the dependent (well‐being) variable was (i) life satisfaction; (ii) happiness; (iii) capability (ICECAP‐O score); and (iv) caring experience (CES) score.

## Results

### Sample characteristics

Of the 1100 carers who responded to the quality‐of‐life survey, 798 (73%) answered the question about whether their decision to care was a free choice. The analyses that follow focus on these individuals. To set the sample of carers in context, Table 1 shows the characteristics of these carers in comparison with all carers who responded to the survey and the noncarer survey respondents. The carers who responded to the questions about choices and constraints were more likely (p < 0.05) to be younger, male, have qualifications, be employed, not be in receipt of benefits and be nonreligious. They were also less likely to care for someone in bad health, be a main carer, care for 50 + hours per week and provide personal care.

**Table 1:** Characteristics of carers responding to the free‐choice question, compared to all carers and noncarers in the sample

Variable	Carers responding to free‐choice question (n = 798)	All carers (n = 1100)	Noncarers (n = 4280)
Socio‐demographic characteristics			
Age (over 65)	21.1%	26.3%	25.9%
Sex (female)	59.8%	61.8%	56.6%
Self‐assessed health			
Good	45.5%	42.3%	49.0%
Fair	40.9%	41.2%	36.8%
Bad	13.5%	16.5%	14.2%
Ethnicity (Black and minority ethnic)	7.1%	7.4%	7.6%
Religious	67.3%	71.2%	62.0%
Formal educational qualifications	77.7%	72.8%	73.8%
Receive means tested benefit	19.3%	21.4%	18.9%
Employed full‐time	31.4%	28.9%	38.7%
Home owner	76.3%	75.6%	72.7%
Care‐related characteristics			
Hours of care			
<20 hours/week	69.4%	65.9%	n/a
20‐49 hours per week	10.0%	10.9%	n/a
50 + hours per week	20.6%	23.2%	n/a
Caring role (main carer)	40.5%	46.2%	n/a
Provide personal care	26.9%	29.5%	n/a
Care recipient health			
Good	18.0%	17.0%	n/a
Fair	45.7%	47.8%	n/a
Bad	36.3%	35.2%	n/a
Care recipient age			
0‐17 years	7.4%	7.4%	n/a
18‐64 years	25.1%	24.3%	n/a
65 years and over	67.6%	68.3%	n/a

Table 2 shows that in terms of the decision to provide care, more than four‐fifths of carers indicated that their decision to provide care was a free choice and over half that they cared out of a sense of ‘duty’. Around a third indicated that no one else was available (39.5%) or that there was no money for paid care (32.5%).

**Table 2:** Choice in the decision to care (n = 1100)

Characteristics of the decision to provide care	Yes	No	No response^a^
I had a free choice to provide care	649	149	302
I provide care because it is my duty	569	256	275
There was no one else to provide care	297	392	411
There was no money for paid care	219	428	453

a the nonrespondents are very highly correlated across questions, so for example, only 28 carers did not answer the free‐choice question, but did subsequently answer the question about money for paid care.

### Perceived choice in caring

**Figure 1:**
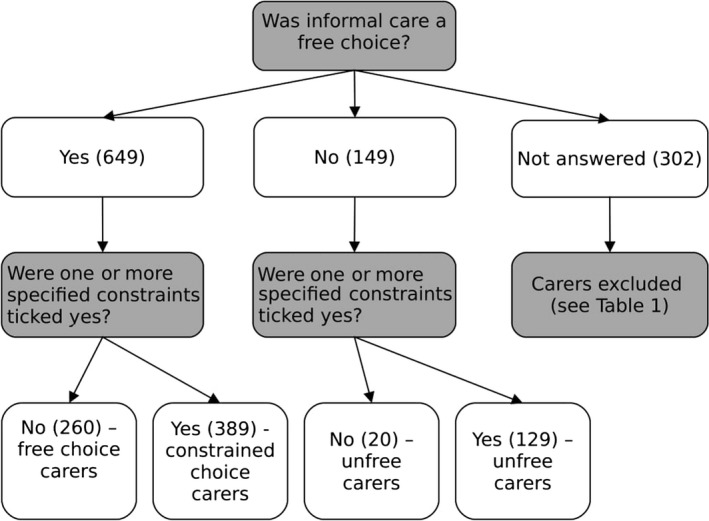
Choice in caring variable (n = 1 100).

Following the creation of the new variable (see Fig. 1), around a third (32.6%) of individuals were ‘free‐choice’ carers. Around half (48.7%) were classified as perceiving a ‘constrained choice’ in caring. Just over a sixth (18.7%) of individuals were classified as ‘unfree’ as they responded negatively to the question on free choice indicating that they did not perceive caring as a free choice. This comprises 16.1% of the sample who indicated that they were ‘constrained’ by duty, a lack of others to care and/or lack of money and 2.5% who responded ‘no’ to the free‐choice question but did not tick any of the options about constraints (unfree and constrained by something other than duty, a lack of others to care or lack of money for paid care).

Table 3 reports the associations between carers’ perceived choice and socio‐demographic characteristics of the carer and the caring role entered into. Carers who perceived themselves to be in bad health were more likely to feel constrained in their caring role (p = 0.03). Also carers who received state benefits were also more likely to see themselves as constrained (p = 0.05). However, none of the other characteristics of the carers were related to the perception of choice in the decision to care (at p < 0.05). In contrast, choice in caring was related to most of the characteristics of the caring role. Perceived choice was lower amongst carers who undertook a primary caring role, provided personal care, cared for 50 + hours per week or cared for someone in bad health. Perceived choice was unrelated to the age of the care recipient. These results indicate that those carers providing the most intensive care (longer hours or personal care and/or in a main caring role caring for someone likely to have more intense care needs) perceived themselves as having the least choice in relation to their caring commitment.

**Table 3:** Associations between individual characteristics and perceived choice in providing informal care (n = 798)

Variable	‘Free‐choice’ carers (n = 260)	‘Constrained choice’ carers (n = 389)	‘Unfree’ carers (n = 149)	Signif. (p‐value)
Socio‐demographic characteristics				
Age (%65 + )	24.4%	19.3%	19.9%	0.46
Sex (% female)	64.6%	52.9%	66.0%	0.75
Health status (% bad)	9.7%	14.3%	17.6%	0.03
Ethnicity (% BME)	3.5%	9.7%	6.4%	0.12
Religious (% yes)	68.1%	71.0%	64.7%	0.52
Qualifications (% yes)	76.6%	78.5%	77.0%	0.87
Means tested benefit (% yes)	15.9%	19.7%	24.6%	0.05
Employed full‐time (% yes)	32.7%	30.3%	32.1%	0.82
Home ownership (% yes)	77.3%	76.0%	76.1%	0.98
Care‐related characteristics				
Hours of care per week (% >50 hours)	7.7%	23.2%	35.8%	<0.01
Caring role (% main carer)	17.9%	46.5%	65.0%	<0.01
Personal care (% providing)	15.6%	29.2%	41.0%	<0.01
Care recipient health (% bad/very bad)	28.8%	34.8%	53.5%	<0.01
Care recipient age (% over 65)	68.6%	70.0%	59.6%	0.53

Significance of associations calculated using Kruskal–Wallis test.

Table 4 shows that carers included in this sample recorded slightly lower levels of well‐being (whether hedonic or eudemonic) on average than noncarers. The mean caring experience in this sample is rated as slightly worse (mean 69 vs 72) than recorded in a recent study of carers of patients at end of life 37.

**Table 4:** Well‐being of carers and noncarers

Well‐being variable	Carers (n = 798)	Non‐carers (n = 4280)
Happiness (% ‘very happy’ or ‘quite happy’)	88.8%	90.4%
Life satisfaction (mean, on 0–10 scale)	7.14	7.35
Capability (mean, on a 0–1 scale)	0.817	0.820
Caring experience (mean, on a 0–100 scale)	69.6	n/a

Table 5 documents associations between well‐being and perceived choice in caring. Across all measures of well‐being, higher levels of choice are associated with higher well‐being. In all cases, the association between well‐being and choice in caring was strongly significant (p < 0.01). Carers who care as a result of a free choice (only) also scored higher than *noncarers* in terms of life satisfaction (p = 0.06), happiness (p = 0.09) and capability (p = 0.02). Conversely, carers who report a lack of free choice in caring report levels of life satisfaction (p < 0.01), happiness (p < 0.01) and capability (p < 0.01) below the level reported by noncarers.

**Table 5:** Associations between well‐being and perceived choice in providing informal care

Variable	‘Free’ carers (n = 260)	‘Constrained’ carers (n = 389)	‘Unfree’ carers (n = 149)	Signif. (p‐value)	Noncarers (n = 4280)
Happiness (% happy)	93.8%	86.4%	81.1%	<0.01	90.4%
Life satisfaction (mean (sd))	7.54 (1.80)	7.07 (1.96)	6.58 (1.85)	<0.01	7.35 (1.94)
Capability (mean (sd))	0.845 (0.10)	0.810 (0.14)	0.783 (0.13)	<0.01	0.820 (0.14)
Caring experience (mean (sd))	75.4 (13.4)	69.9 (16.5)	61.2 (17.7)	<0.01	n/a

Significance of associations calculated using Kruskal–Wallis test.

The regression models express well‐being in terms of carers’ life satisfaction, capability and caring experience (Table 6) and carers’ happiness (Table 7). The reported regressions in Table 6 were estimated using ordinary least squares (OLS). As a sensitivity analysis, we also estimated the life satisfaction model using ordered logit and the carers’ capability model using a double‐censored Tobit (censored at 0 and 1). The OLS regression models confirm that the strong association between choice in caring and well‐being persists when controlling for the presence of other contextual variables relating to the characteristics of the carer and the caring situation. The results from sensitivity analyses (available on request) also demonstrate the same pattern of strong association between freedom of choice and carer well‐being, albeit with some minor differences in the significance of contextual variables.

**Table 6:** OLS regression models of the association between well‐being and free choice in caring (n = 798)

Independent variable	MODEL 1 Life satisfaction 1‐10 scale	MODEL 2 Capability well‐being 0‐1 scale	MODEL 3 Caring experience 0‐100 scale
Socio‐demographic variables			
Age (65 + )	0.79 (0.20)***	−0.005 (0.013)	−0.8 (2.0)
Sex (female)	0.26 (0.15)	0.004 (0.009)	2.3 (1.4)
Health (bad)	−1.14 (0.21)***	−0.107 (0.013)***	−8.5 (1.9)***
Ethnicity (BME)	−0.58 (0.29)*	−0.044 (0.018)*	−4.5 (2.6)
Religious (yes)	0.27 (0.15)	0.020 (0.009)*	1.4 (1.4)
Qualifications (yes)	0.33 (0.18)	−0.012 (0.011)	−4.1 (1.7)*
Means tested benefit (yes)	−0.36 (0.19)	−0.026 (0.012)*	−10.0 (1.8)***
Employed full‐time (yes)	0.27 (0.16)	0.005 (0.010)	0.2 (1.5)
Home ownership (yes)	0.35* (0.17)	0.042 (0.011)***	2.6 (1.6)
Care‐related variables			
Caring hours (50 hours +)	−0.14 (0.22)	−0.003 (0.014)	−2.6 (2.0)
Caring role (main carer)	−0.14 (0.17)	−0.008 (0.011)	−5.4 (1.6)***
Personal care (provided)	−0.18 (0.17)	0.007 (0.011)	0.7 (1.5)
Care recipient health (bad)	−0.12 (0.15)	−0.009 (0.009)	−2.9 (1.4)*
Care recipient age (over 65)	0.06 (0.12)	0.014 (0.007)*	−1.6 (1.1)
Choice in caring			
Unfree	omitted	omitted	omitted
Constrained	0.45 (0.19)*	0.019 (0.012)	6.7 (1.7)***
Free	0.72 (0.22)***	0.047 (0.013)***	10.4 (2.0)***
R^2^	0.156	0.210	0.254
Sample size (n)	688	679	568

*p < 0.05; **p < 0.01; ***p < 0.001.

Cell values represent the beta coefficients in the regression models, with standard errors in parentheses.

**Table 7:** Ordered logit regression model of the association between happiness and free choice in caring (n = 798)

Independent variable	MODEL 4 Happiness 1, 2, 3, 4
Socio‐demographic variables	
Age (65 + )	0.81 (0.19)
Sex (female)	0.81 (0.14)
Health (bad)	4.06 (1.06)***
Ethnicity (BME)	2.44 (0.88)*
Religious (yes)	0.69 (0.12)*
Qualifications (yes)	1.03 (0.22)
Means tested benefit (yes)	1.62 (0.15)*
Employed full‐time (yes)	1.08 (0.21)
Home ownership (yes)	0.50 (0.11)***
Care‐related variables	
Caring hours (50 hours +)	1.18 (0.31)
Caring role (main carer)	1.24 (0.25)
Personal care (provided)	1.21 (0.17)
Care recipient health (bad)	1.02 (0.18)
Care recipient age (over 65)	0.95 (0.13)
Choice in caring	
Unfree	omitted
Constrained	0.68 (0.16)
Free	0.43 (0.11)***
Pseudo R^2^	0.081
Sample size (n)	688

*p < 0.05; **p < 0.01; ***p < 0.001.

Cell values represent odds of appearing in a worse happiness state, with standard errors in parentheses.

Controlling for other factors, having a constrained choice in caring (relative to no free choice) was associated, on average, with the following:

- Higher life satisfaction (nearly half a point, on a 1–10 scale);
- Higher capability (0.02 points on a 0–1 scale);
- Better caring experience (6.7 points on a 0‐100 caring experience scale);
- Lower odds of reporting being unhappy (odds ratio 0.68).

Controlling for other factors, having a free and unconstrained choice in caring (relative to no free choice) was associated with even higher life satisfaction, capability, caring experience and happiness. The magnitude of these associations with well‐being can be put into context by comparing them with other factors associated with well‐being. For example, in terms of life satisfaction, the positive impact of free (unconstrained) choice is greater than the positive impact of having educational qualifications or home ownership. In terms of capability, the positive impact of free choice is comparable to the positive association with home ownership.

## Discussion

This study suggests that people often see participation in informal care as *both* a choice and a constraint. In this survey, around half the sample of UK carers described their decision to provide informal care as a free choice but constrained by a sense of duty, financial resource or lack of social support. Most socio‐demographic factors were not related to the perception of choice in caring. Conversely, aspects of the caring role linked to caring intensity (such as being a primary carer and providing personal care) were strongly associated with a perception of less choice. The perception of choice was strongly related to carers’ subsequent well‐being, controlling for the fact that those carers who perceived free choice tended to have less intensive caring roles.

The finding that many people perceived some form of constraint in caring is not surprising, given previous research 17, 23, 38. This corresponds with other studies in the UK and the Netherlands 17, 22, which suggest that duty and an expectation that care will be provided are prime factors behind the decision to care. However, the high proportion of carers who felt their decision was a free choice even though constraints were present is more intriguing. Viewed from the rational choice perspective, which is based on the notion of a human being weighing up cost benefits of his or her decisions, this could be seen as reflecting the complex reality of the decision to care. It is consistent, for example, with the carer needing to make a series of decisions regarding the choice to care in the face of a range of constraints. People may enter and exit caring, and make decisions about how much care to provide and what tasks they can undertake. There may be an element of choice in some aspects of these decisions and not in others. For instance, Arksey and Glendinning 8 draw a distinction between the decision to enter a caring role and the choices within the caring role. Furthermore, decisions about caring are neither made in a vacuum nor at a single moment in time, and therefore, scope for choice about caring can vary over life courses, for example because of age and wider social factors including changing gender norms 39, 40 as well as gaps and transitions in careers, family environments 41 and other more temporally proximal events 42.

It is also possible that while the decision to take on caring is constrained by economic, social and normative pressures and might not appear by others to be chosen, nonetheless, the person taking on this role benefits from *perceiving* it as being chosen from free will. Psychological theory and research suggests and demonstrates that people are meaning‐making beings and that subjective meanings often mediate between a situation and a person's reaction to it 43. Having a sense of perceived control is vitally connected to well‐being 44, and generating a belief that one has entered into caregiving through choice is a protective coping strategy that is likely to enable a carer to continue with their role without resentment 45. It may be much better for a person's health to see himself or herself as having taken on caring not due to societally imposed duty, but due to his or her internalised values about the importance of looking after close family members. It may be the value of perceived control that is responsible for the majority of carers in our sample falling into the group of those with ‘constrained choice’.

The lack of association between perceived choice and socio‐demographic characteristics was unexpected. We did not find that perceived choice was any lower, for example, for women or those with less financial capability. This may be because the perception of choice to provide care is highly subjective. It might also be because choice in caring is more strongly influenced by other factor. For example, studying the kin relationship may reveal less perceived freedom in the decision to undertake spousal or parental care than care for an elderly relative or friend. Likewise studying the degree of prior attachment may help explain why a daughter who feels close to her elderly mother may choose to take on the role of caring, over and above other siblings who do not feel as emotionally close.

We also found that choice in caring was strongly linked with the entry into secondary caring roles, especially where the recipient was not in ‘bad’ health and when no personal care was provided. One interpretation is that the perception of choice about the decision to provide care is evaluated in the context of the intensity of ongoing care provision, as much as in relation to the initial decision to provide care. In situations where a care role is less intensive, the carer takes on the role knowing he or she retains some freedom to live life other than through caring. In circumstances where care needs are more intensive, becoming the primary carer will constrain or force other roles to be abandoned or neglected. Furthermore, the carer may not be in a position to withdraw if the one cared for is in poor health. In these circumstances, it becomes harder to generate a narrative of choice, hence the association of lower choice with being a primary carer, providing personal care and caring for someone in poor health.

While a positive relationship between choice in caring and well‐being was expected, the magnitude of the effect found in this study is worth emphasising. Choice in caring seems as important in terms of the carer's well‐being, if not more so, than more tangible factors, for example whether the carer provides personal care or whether the care recipient is in good health. Choice in caring may therefore be a valuable target for policymakers concerned about improving carer's well‐being. Future research might focus on developing an understanding of the degree to which social policy can expand perceived choice in caring and which aspects of choice can and ought to be targeted. It is important to highlight that choice is important not only in entry to the caring role, but also within the caring role. As a result, there may be gains to carers from expanding choice in the amount and type of social care support 46 and combining work and care 8, 47. Interventions for carers of people with long‐term conditions include elements aimed at increasing perceived control and capability 48. Further development and evaluation of such approaches through media other than 1 : 1 therapy, such as social media or self‐help networks, could be a further way forward.

It is important, however, not to draw definitive conclusions about a causal link between choice in caring and well‐being. This is a cross‐sectional study, and it could be the case that carers with better well‐being were more able to exercise choice about whether to take on a caring role, or their higher well‐being made them more likely to feel as if they had choice to take on the role. Furthermore, the lack of some key variables means that we are unable to describe the sample as comprehensively as we would have liked, and examine other potentially relevant factors in relation to carers’ choices and constraints. A range of factors, in addition to duty, family support and financial resources, are likely to motivate and constrain decisions in relation to providing informal care. For example, we did not explore some of the positive motivations for care, such as emotional bonds, that have been highlighted in other studies 24, 25. Finally, a limitation of this work is the low response rates to both the quality‐of‐life survey as whole and the questions about choices and constraints in caring. Some carers may have found these questions difficult to answer or intrusive. This may have created some selection bias; for example, carers who responded to these questions were more likely to be in higher socio‐economic groups than those who did not answer the questions and less likely to be in intensive caring roles. When considering the generalisability of the results, it is also important to note that the analysis is limited to a sample of carers living in one city in England.

## Conclusion

This study suggests that, for many, entry into caring is perceived as both a choice and a constraint. Perception of choice in entering caring is positively associated with well‐being. Further research is needed to specify aspects of caring where free choice generates higher well‐being and whether these can be enhanced by social policy. Nevertheless, this study suggests, in general terms, that there may be significant benefits to carers from enhancing their choice on entry to a caring role.

## Author contributions

HA conducted the data analysis and wrote the first draft of the manuscript. All authors contributed to the study design, study conduct and later drafts of the manuscript.

## Ethical approval

The study is based on data collected as part of a local government survey of quality of life of local residents. The local government organisation has an internal ethical review process for their survey work.

## Funding

Hareth Al‐Janabi was supported through this work by an MRC early career fellowship in economics of health (G1002334).

## Supporting information


**Appendix S1.** Key questions from the Quality of Life survey
