# Supplementary material for: Informal care: choice or constraint?
Source: Scand J Caring Sci. 2017 Apr 12;32(1):157–67. doi: 10.1111/scs.12441 (PMC5873411; doi:10.1111/scs.12441)
Supplement: Supplementary file 1 — Appendix S1. Key questions from the Quality of Life survey [file SCS-32-157-s001.doc]

**Supplementary material: Key questions from the Quality of Life survey**

**Identification of carers**

Do you look after, or give any help or support to family members, friends, neighbours or others because of long-term physical or mental ill-health or disability, or problems related to old age?” (Please tick time spent in a typical week. Do not count anything you do as part of your paid employment)

No

Yes, 1-19 hours per week

Yes, 20-49 hours per week

Yes, 50+ hours per week

**Decision to care**

Thinking about your decision to provide care, please tick ‘yes’ or ‘no’ to the following questions:

I provide care because it is my duty

I had a free choice to provide care

There was no-one else to provide care

There was no money to provide care

**Happiness**

Taking all things together, would you say you are? (Please tick 1 box)

Very happy

Quite happy

Not very happy

Not at all happy

**Life satisfaction**

All things considered, how satisfied are you with your life as a whole these days? (Please circle a number: 1 means you are completely dissatisfied and 10 means you are completely satisfied)

1 2 3 4 5 6 7 8 9 10

**ICECAP-O (capability) items**

Love and friendship (able to have …all/ a lot / a little/none)

Thinking about the future (able to…without any/only a little/with some/ with a lot of… concern)

Doing things that make you feel valued (able to do …all/many/a few/none)

Enjoyment and pleasure (can have …all/ a lot/ a little/ none)

Independence (able to be independent…completely/in many things/ in a few things/no things)

**Carer Experience Scale items**

Activities outside caring (most/some/few)

Support from family and friends (a lot/some/a little)

Assistance from organisations and the government (a lot/some/a little)

Fulfilment from caring (mostly/sometimes/rarely)

Control over aspects of caring (most/some/ a few)

Getting on with the person you care for (mostly/sometimes/rarely)

The full ICECAP-O and Carer Experience Scale questionnaires are available at [www.icecap.bham.ac.uk/](http://www.icecap.bham.ac.uk/).
